# A U-shaped relationship between the atherogenic index of plasma and repeated target vessel revascularization in patients undergoing percutaneous coronary intervention: a retrospective study

**DOI:** 10.3389/fendo.2024.1428830

**Published:** 2024-11-11

**Authors:** Xingjie Huang, Song Wen, Zehan Huang, Guishi Qin, Hanli Zhou, Zhonghua Xia

**Affiliations:** ^1^ Department of Cardiology, The Second Affiliated Hospital of Guilin Medical University, Guilin, Guangxi, China; ^2^ Department of Cardiology, Guangdong Cardiovascular Institute, Guangdong Provincial People’s Hospital, Guangdong Academy of Medical Sciences, Southern Medical University, Guangzhou, Guangdong, China; ^3^ Scientific Research Department, Liuzhou Worker’s Hospital, Liuzhou, Guangxi, China

**Keywords:** atherogenic index of plasma, coronary artery disease, percutaneous coronary intervention, drug-eluting stents, target vessel revascularization

## Abstract

**Background:**

Atherogenic index of plasma (AIP) has been recognized as a novel and practical marker for the assessment of cardiometabolic risk, but the relevance of AIP as a prognostic biomarker in coronary artery disease (CAD) remains debated. This study investigated the association between AIP and major adverse cardiac and cerebrovascular events (MACCEs) in CAD patients receiving percutaneous coronary intervention (PCI) with drug-eluting stents (DES).

**Methods:**

A total of 2,250 patients undergoing PCI with DES were included in this retrospective cohort study. The primary endpoint was MACCEs, encompassing acute myocardial infarction, repeat target vessel revascularization (TVR), stroke, and all-cause mortality. Logistic regression models were used to estimate odds ratios (ORs) and 95% confidence intervals (CIs). Restricted cubic splines were applied to explore the dose–response associations. And subgroup analysis was conducted to evaluate potential relationship between AIP and MACCEs across different subgroups.

**Results:**

During a medium follow-up of 29.8 (25.6–34.0) months, 106 (4.7%) patients experienced TVR. After adjusting for confounders, AIP (per 1 SD increase) was positively associated with TVR (odds ratio [OR] = 1.26, 95% confidence interval [CI] = 1.01–1.58, *P* = 0.042). In females, there was a significant association (OR = 2.33, 95% CI = 1.40–3.98, *P* = 0.002), but no significant association was observed in males. There was an interaction between AIP and gender (*P* = 0.017). Restricted cubic spline analysis depicted a U-shaped relationship between AIP and TVR (*P*_nonlinear_ = 0.016), with an elevated risk evident from an AIP of 0.20.

**Conclusion:**

AIP showed a U-shaped relationship with TVR in PCI patients with DES, particularly pronounced among females. We suggested that the AIP should be used as a plasma marker of key interest for preventing TVR after DES implantation in patients with CAD.

## 1 Introduction

Coronary artery disease (CAD) is increasingly becoming a significant global health challenge due to its rising prevalence (1, 2). Percutaneous coronary intervention (PCI) is a primary therapeutic strategy for management CAD, with drug-eluting stents (DES) offering distinct advantages over bare-metal stents (3). However, the use of second-generation DES can lead to complications such as in-stent thrombosis, restenosis, and major adverse cardiovascular events (MACEs), affecting patient outcomes (4, 5). Factors influencing MACEs post-stenting include abnormal lipid metabolism, a common etiological factor (6). Lipid-lowering therapy post-PCI, particularly targeting low-density lipoprotein cholesterol (LDL-C), is crucial for improving long-term patient outcomes (7). Despite adherence to guidelines for lipid-lowering treatment, residual cardiovascular risk persists, likely due to dyslipidemic profiles characterized by elevated triglycerides (TG) and reduced high-density lipoprotein cholesterol (HDL-C) in post-PCI patients (8, 9).

The atherogenic index of plasma (AIP), calculated as log10(TG/HDL-C), is a novel indicator of CAD risk and insulin resistance-related disorders (10–13). It predicts atherosclerosis severity, coronary artery lesions, calcification, plaque progression, and chronic total occlusions (CTO) (14–18). Several studies have shown that the AIP is a strong indicator of worse outcomes in patients with CAD, however this finding is still debated (19–23). Most researches focused on CAD patients undergoing PCI or those with acute coronary syndrome (ACS) and comorbid diabetes mellitus (DM) (19–22). Since PCI often involves patients with stable angina (SA) and ACS with various comorbidities, assessing AIP for predicting major adverse cardiac and cerebrovascular events (MACCEs) post-DES is clinically relevant. Limited research exists on the predictive value of AIP for MACCEs in Chinese CAD patients post-DES. This study aimed to evaluate the relationship between AIP and MACCEs incidence, providing clinical insights.

## 2 Methods

### 2.1 Study design and population

Data were sourced from the Dryad database (https://doi.org/10.5061/dryad.13d31), provided by Yao HM et al. (24, 25). The study included 2,533 patients who underwent PCI with DES at the First Affiliated Hospital of Zhengzhou University (July 2009 to August 2011).

Coronary angiography and PCI were conducted per standard guidelines. Preoperatively, patients received aspirin 300 mg and clopidogrel 300 mg unless they were on prior antiplatelet therapy. The choice of therapeutic strategy, stent type, glycoprotein IIb/IIIa inhibitors, and intravascular ultrasound was at the operator’s discretion. Post-surgery, patients were prescribed dual antiplatelet therapy (100 mg/day aspirin and 75 mg/day clopidogrel) for at least a year, barring contraindications. Follow-up had a median duration of 29.8 (25.6–34.0) months.

### 2.2 Patient screening and selection

**Figure 1:**
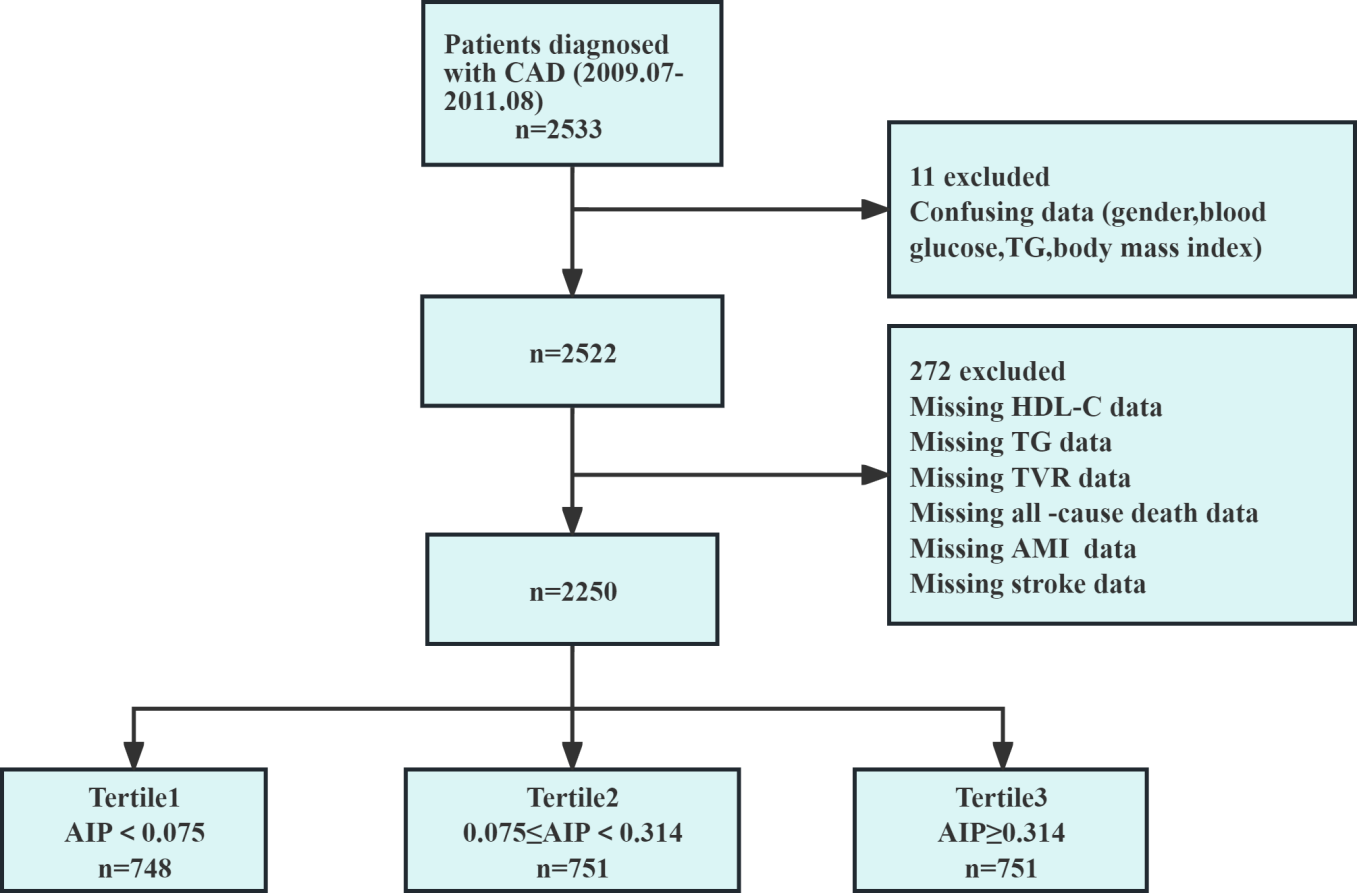
Flow chart of the study population. AIP, atherogenic index of plasma; AMI, acute myocardial infarction; CAD, coronary artery disease; HDL-C, high-density lipoprotein cholesterol; n, number of patients; TG, triglyceride; TVR, target vessel revascularization.

**Figure 1** showed the patient screening process. Initially, 2,533 CAD patients were considered. Exclusions were based on confusing data for gender, blood glucose, TG, and BMI (n=11), or incomplete HDL-C and TG data, and those lost to follow-up (n=272). Finally, 2,250 patients were included. The study received ethical approval and informed consent waiver from the Ethics Committee of the First Affiliated Hospital of Zhengzhou University, aligning with the Declaration of Helsinki.

### 2.3 Data collection and definitions

Demographic data, including age, gender, and BMI at the time of admission, were routinely collected from patients. The documented medical history included heart failure (HF), atrial fibrillation (AF), DM, hypertension, old myocardial infarction (OMI), stroke, prior PCI, prior coronary artery bypass grafting (CABG), and a history of smoking. Fasting blood samples assessed laboratory parameters: glucose, creatinine, uric acid (UA), bilirubin, LDL-C, total cholesterol (TC), HDL-C, and TG. Details on coronary angiography and DES specifics were recorded, including surgical approach, diseased vessel characteristics, and stent details. Medications during hospitalization were documented. Data were retrieved from the electronic medical record system and follow-up information via outpatient visits, calls, and readmission records.

### 2.4 Calculations and definitions

The AIP was defined as log10(TG/HDL-C). Smoking history was defined per WHO guidelines as continuous smoking for the previous 10 years. Diabetes and hypertension histories were defined by self-reported diagnosis by a physician or current medications usage for these conditions. CAD included SA, non-ST-elevation acute coronary syndrome (NSTE-ACS), and ST-elevation myocardial infarction (STEMI). Target vessel revascularization (TVR) was defined as repeated revascularization of the initial target vessel during follow-up, via either PCI or CABG.

### 2.5 Clinical endpoint

The primary endpoint was major adverse cardiac and cerebrovascular events (MACCEs): all-cause mortality, acute myocardial infarction, TVR, and stroke.

### 2.6 Statistical analysis

**Figure 2:**
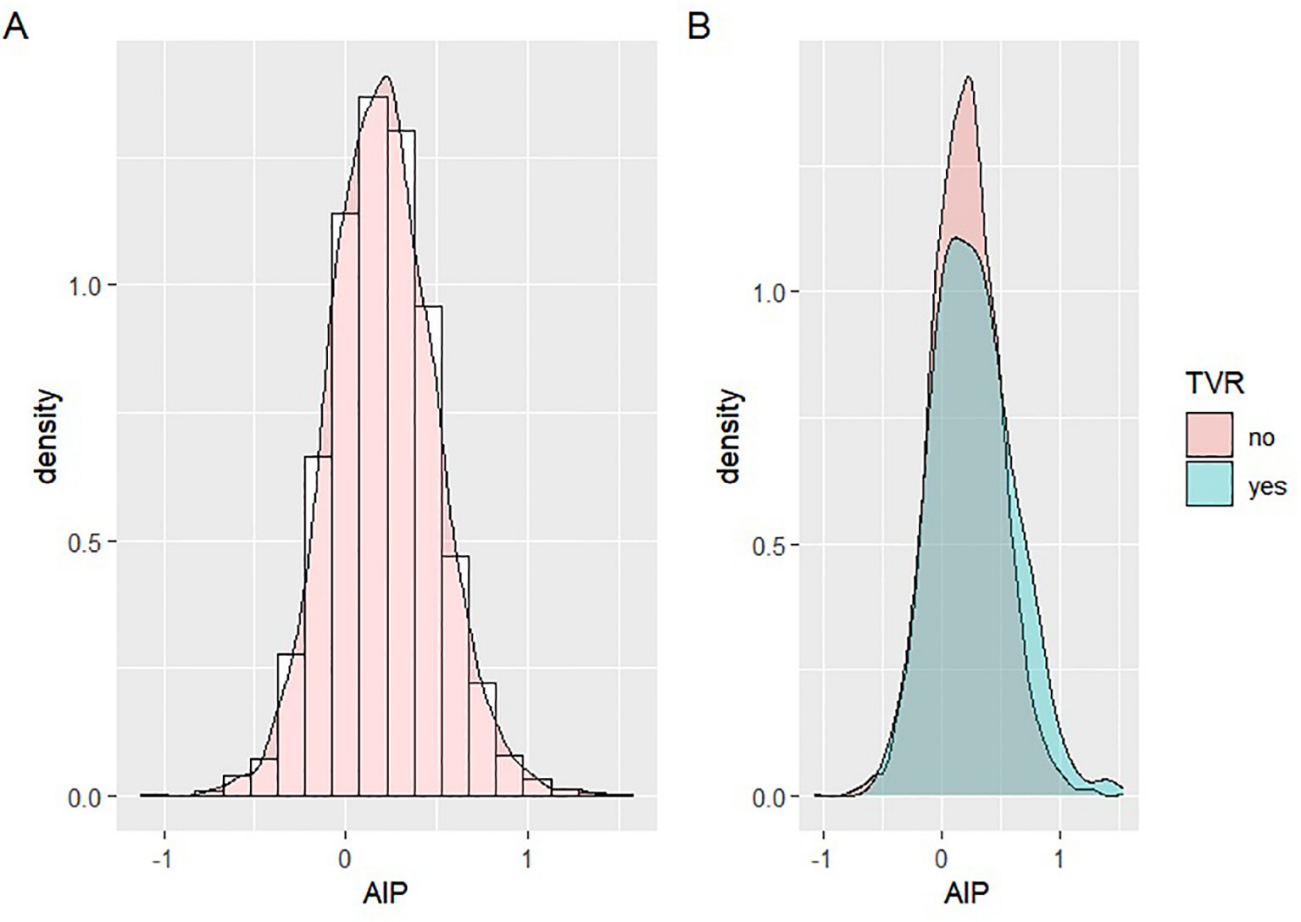
Distribution of AIP data. **(A)** Distribution of AIP in the overall population; **(B)** Distribution of TVR in the TVR group and the non-TVR group.

Patients were stratified by AIP tertiles: Tertile 1 (AIP < 0.075), Tertile 2 (0.075 ≤ AIP < 0.314), and Tertile 3 (AIP ≥ 0.314). AIP distribution was assessed using histograms and probability density plots (**Figure 2**), showing a distribution that approximated normality across the general population, as well as in the TVR and non-TVR subgroups. The baseline characteristics of these groups were outlined. Categorical variables were presented as frequencies (n) and percentages (%), and comparisons among groups were conducted using the Chi-square test. Continuous variables were reported as means ± standard deviations (SD), with the t-test applied to pairwise comparisons of normally distributed data and one-way ANOVA for multiple group comparisons. The Kruskal-Wallis test was used to analyze non-normally distributed data, which was shown as interquartile ranges (IQR).

Z-score was used to standardize AIP values, which were then included in univariable and multivariable logistic regression models to assess the effect of each SD increment in AIP on MACCEs and TVR. The analysis was further refined through three models to ascertain the association between AIP and TVR. Model 1 was unadjusted; Model 2 was adjusted for age and gender; and Model 3 was extensively adjusted for a comprehensive set of variables including age, gender, systolic/diastolic blood pressure (SBP/DBP), stroke, prior PCI, smoking, AF, OMI, hypertension, DM, CAD, three-vessel disease, restenosis, CTO, bifurcation lesions, number of treated vessels, type of stents, diameter of stents, length of stents, involvement of the left main coronary artery (LM), right coronary artery (RCA), left anterior descending artery (LAD), LDL-C, UA, blood glucose, and medication use (aspirin, clopidogrel, and statins). Variables were selected based on a variance inflation factor greater than 10, a change in the regression coefficient of AIP by more than 10%, a *p*-value less than 0.05 in the regression coefficient for TVR, and clinical relevance. Logistic regression results were expressed as odds ratios (ORs) with 95% confidence intervals (CIs).

Subgroup analyses were performed to examine possible interactions between AIP and variables such as gender, age, hypertension, CAD, DM, and LDL-C levels. The potential nonlinear relationship between AIP and TVR was examined using restricted cubic spline (RCS) analysis, selecting three knots at the 10th, 50th, and 90th percentiles. Statistical significance was determined by a two-sided *p*-value of <0.05. All statistical analyses were performed using R software version 4.2.2 and IBM SPSS Statistics version 25.

## 3 Results

### 3.1 AIP and clinical outcomes in the overall population

A total of 2,250 patients were enrolled in the study, comprising 1,532 males and 718 females with a mean age of 60.0 ± 11.1 years. **Table 1** presented the clinical characteristics of the participants. Demographically, the higher AIP groups were characterized by a younger age, a greater proportion of males, and elevated SBP/DBP (all *p* < 0.05). Clinically, these groups exhibited a higher prevalence of DM, hypertension, and smoking (all *p* < 0.05). Pharmacologically, higher AIP groups used less aspirin and more beta-blockers (*p* < 0.05). Laboratory analyses revealed that TG, TC, LDL-C, and bilirubin were significantly elevated in patients with higher AIP, whereas levels of blood glucose, uric acid, and HDL-C were significantly lower (all *p* < 0.05).

**Table 1:** Baseline characteristics of the study population.

Characteristic	Overall N = 2,250	Tertile 1 N = 748	Tertile 2 N = 751	Tertile 3 N = 751	*p*-value
Demographics					
**Age, years**	60.0±11.1	62.7±10.7	60.0±10.7	57.3±11.1	<0.001
**Male,n (%)**	1,532 (68%)	466 (62%)	527 (70%)	539 (72%)	<0.001
**BMI, kg/m^2^**	23.9±3.8	23.7±3.7	23.7±3.8	24.2±3.9	0.073
**SBP,mmHg**	103±29	100±29	104±29	104±29	0.005
**DBP,mmHg**	77±12	76±13	77±11	78±12	<0.001
Medical history					
**HF, n(%)**	269 (12%)	95 (13%)	87 (12%)	87 (12%)	0.747
**AF, n(%)**	44 (2.0%)	19 (2.5%)	15 (2.0%)	10 (1.3%)	0.237
**OMI, n(%)**	221 (9.8%)	81 (11%)	75 (10.0%)	65 (8.7%)	0.361
**Stroke, n(%)**	118 (5.2%)	43 (5.7%)	38 (5.1%)	37 (4.9%)	0.746
**Prior PCI, n(%)**	149 (6.6%)	61 (8.2%)	44 (5.9%)	44 (5.9%)	0.120
**Prior CABG, n(%)**	16 (0.7%)	7 (0.9%)	3 (0.4%)	6 (0.8%)	0.438
**Hypertension, n(%)**	1,133 (50%)	351 (47%)	371 (49%)	411 (55%)	0.009
**DM, n(%)**	489 (22%)	117 (16%)	167 (22%)	205 (27%)	<0.001
**Smoking, n(%)**	740 (33%)	197 (26%)	264 (35%)	279 (37%)	<0.001
**CAD, n(%)**					0.374
STEMI	564 (25%)	183 (24%)	179 (24%)	202 (27%)	
NSTE-ACS	1,365 (61%)	449 (60%)	461 (61%)	455 (61%)	
SA	321 (14%)	116 (16%)	111 (15%)	94 (13%)	
Laboratory data					
**Blood glucose (mmol/L)**	5.2 (4.7, 6.3)	5.1 (4.6, 6.0)	5.1 (4.6, 6.1)	5.4 (4.8, 6.8)	<0.001
**Creatinine (µmol/L)**	69.0 (58.0, 81.0)	68.0 (56.0, 81.0)	70.0 (58.9, 82.0)	70.0 (58.2, 81.0)	0.077
**UA(µmol/L)**	294.0 (244.0, 353.0)	271.0 (225.0, 334.0)	298.0 (244.0, 352.5)	312.0 (266.0, 372.0)	<0.001
**Bilirubin (umol/L)**	8.6 (6.0, 12.0)	8.7 (6.3, 12.3)	8.9 (6.2, 12.2)	8.2 (5.8, 11.3)	0.001
**HDL-C (mmol/L)**	1.1±0.3	1.3±0.3	1.0±0.2	0.9±0.2	<0.001
**TG (mmol/L)**	1.58 (1.14, 2.25)	1.03 (0.83, 1.23)	1.56 (1.32, 1.87)	2.69 (2.12, 3.49)	<0.001
**TC (mmol/L)**	4.3±1.1	4.1±1.0	4.2±1.0	4.5±1.1	<0.001
**LDL-C (mmol/L)**	2.7±0.9	2.6±0.9	2.7±0.9	2.7±1.0	0.002
Treatment					
**aspirin, n(%)**	2,220 (99%)	738 (99%)	748 (100%)	734 (98%)	0.019
**Clopidogrel, n(%)**	2,158 (96%)	716 (96%)	712 (95%)	730 (97%)	0.060
**statin, n(%)**	2,111 (94%)	698 (93%)	716 (95%)	697 (93%)	0.098
**Beta blocker, n(%)**	1,583 (70%)	502 (67%)	547 (73%)	534 (71%)	0.045
**ACEI, n(%)**	1,248 (55%)	400 (53%)	419 (56%)	429 (57%)	0.342
**CCB, n(%)**	557 (25%)	176 (24%)	189 (25%)	192 (26%)	0.626
**Radial artery access, n(%)**	2,195 (98%)	730 (98%)	734 (98%)	731 (97%)	0.879
Number of diseased vessels					
1-vessel disease	877 (39%)	308 (41%)	296 (39%)	273 (36%)	0.153
2-vessel disease	836 (37%)	264 (35%)	288 (38%)	284 (38%)	0.426
3-vessel disease	532 (24%)	175 (23%)	166 (22%)	191 (25%)	0.310
Characteristics of lesions					
CTO**, n(%)**	197 (8.8%)	66 (8.8%)	67 (8.9%)	64 (8.5%)	0.960
occulsion, n(%)	296 (13%)	92 (12%)	111 (15%)	93 (12%)	0.272
Bifurcation lesion, n(%)	396 (18%)	145 (19%)	126 (17%)	125 (17%)	0.291
restenosis, n(%)	29 (1.3%)	13 (1.7%)	6 (0.8%)	10 (1.3%)	0.271
Location of target lesions					
LM, n(%)	72 (3.2%)	29 (3.9%)	19 (2.5%)	24 (3.2%)	0.334
LAD, n(%)	1,859 (83%)	635 (85%)	614 (82%)	610 (81%)	0.129
LCX, n(%)	1,096 (49%)	354 (47%)	362 (48%)	380 (51%)	0.422
RCA, n(%)	1,109 (49%)	355 (47%)	367 (49%)	387 (52%)	0.277
CABG, n(%)	2 (<0.1%)	1 (0.1%)	1 (0.1%)	0 (0%)	0.777
**Number of treated vessels**					0.358
1, n(%)	1,293 (57%)	438 (59%)	438 (58%)	417 (56%)	
2, n(%)	757 (34%)	237 (32%)	256 (34%)	264 (35%)	
≥3, n(%)	200 (8.9%)	73 (9.8%)	57 (7.6%)	70 (9.3%)	
**Diameter of stents, mm**	3.10±0.91	3.07±0.44	3.14±1.46	3.08±0.44	0.233
**Length of stents, mm**	42.0 (24.0, 66.0)	39.0 (23.0, 66.0)	42.0 (24.0, 69.0)	42.5 (25.0, 66.0)	0.084
**Type of stents**					0.530
Sirolimus-eluting stent, n(%)	1,464 (65%)	489 (65%)	503 (67%)	472 (63%)	
paclitaxel-eluting stent, n(%)	443 (20%)	141 (19%)	144 (19%)	158 (21%)	
Other drug-eluting stents, n(%)	339 (15%)	117 (16%)	104 (14%)	118 (16%)	
**MACCEs**, n(%)	346 (15%)	128 (17%)	105 (14%)	113 (15%)	0.232
**All-cause death**, n(%)	165 (7.3%)	66 (8.8%)	52 (6.9%)	47 (6.3%)	0.142
**AMI**, n(%)	103 (4.6%)	40 (5.3%)	30 (4.0%)	33 (4.4%)	0.436
**Stroke**, n(%)	36 (1.6%)	15 (2.0%)	8 (1.1%)	13 (1.7%)	0.328
**TVR**, n(%)	106 (4.7%)	33 (4.4%)	28 (3.7%)	45 (6.0%)	0.104

Categorical variables were expressed as frequencies (n) and percentages (%).

N, number of patients; AMI, acute myocardial infarction; MACCEs, major adverse cardiac and cerebrovascular events; TVR, target vessel revascularization.

ACEI, angiotensin converting enzyme inhibitors; AF, atrial fibrillation; BMI, body mass index; CAD, coronary artery disease; CABG, coronary artery bypass grafting; CCB, calcium channel blocker; CTO, chronic total occlusions; DBP, diastolic blood pressure; DM, diabetes mellitus; HDL-C, high-density lipoprotein cholesterol; HF, heart failure; LAD, left anterior descending; LM, left main coronary artery; LCX, left circumflex artery; LDL-C, low-density lipoprotein cholesterol; N, number of patients; NSTE-ACS, non-ST-segment elevation acute coronary syndromes; OMI, old myocardial infarction; PCI, Percutaneous coronary intervention; RCA, right coronary artery; STEMI, ST-segment elevation myocardial infarction; SA, stable angina; SBP, systolic blood pressure; TC, total cholesterol; TG, triglyceride.

**Table 1** (continued) showed that in the comparison among groups divided into tertiles based on AIP, no significant differences were observed in MACCEs, all-cause death, AMI, and stroke. However, the risk of TVR was lowest in the second tertile group, with higher risks in both the first and third tertile groups, showing a “U”-shaped change in the risk of TVR across the three groups (4.4% vs. 3.7% vs. 6.0%, *p* = 0.104).

### 3.2 Risk factors of MACCEs

We used logistic regression analysis to identify the risk factors for MACCEs in the study population. First, univariable analysis of covariance between the respective variables and MACCEs was performed, followed by testing the covariance between the other variables and the AIP. TC, TG, and HDL-C were excluded from the final analysis. Age, HF, AF, OMI, prior PCI, stroke, DM, SBP, creatinine, UA, angiotensin-converting enzyme inhibitor(ACEI), 1-vessel disease, 3-vessel disease, CTO, RCA, LCX, LAD, number of treated vessels, diameter of stents, length of stents, and type of stents were included in the multivariable regression analysis. The results suggested that age, HF, CTO, SBP, number of treated vessels, and diameter of stents were independently associated with MACCEs (*p* < 0.05). However, the AIP (per 1 SD increase) and MACCEs were not significantly associated in both univariable analysis (OR = 0.98, 95% CI = 0.87–1.10, *p* = 0.693) and multivariable analysis (OR = 1.03, 95% CI = 0.90-1.17, *p* = 0.694) (**Table 2**).

**Table 2:** Univariable and Multivariable analysis for predictors of MACCEs.

	Univariate analysis			Multivariate analysis		
Characteristic	OR	95% CI	p-value	OR	95% CI	p-value
AIP (per 1 SD increase)	0.98	0.87, 1.10	0.693	1.03	0.90, 1.17	0.694
Age	1.047	1.034, 1.06	<0.001	1.04	1.03, 1.05	<0.001
Sex	0.96	0.75, 1.22	0.746			
BMI	1.03	0.99,1.07	0.164			
HF	1.9	1.39, 2.56	<0.001	1.63	1.17, 2.26	0.004
AF	2.10	1.07, 4.12	0.031	1.69	0.80, 3.34	0.146
OMI	1.61	1.14, 2.21	0.006	1.26	0.86, 1.82	0.226
stroke	1.87	1.20, 2.88	0.005	1.50	0.92, 2.38	0.090
Prior PCI	1.49	0.98, 2.25	0.059	1.56	0.99, 2.41	0.058
Prior CABG	1.84	0.59, 5.75	0.291			
Hypertension	1.21	0.96, 1.52	0.109			
DM	1.42	1.09, 1.85	0.008	1.22	0.91, 1.62	0.173
Smoking	1.06	0.84,1.36	0.601			
SBP	1.004	1.0002, 1.0082	0.037	1.01	1.00, 1.01	0.018
DBP	1.01	0.99, 1.02	0.127			
CAD						
STEMI	-	-				
NSTE-ACS	0.97	0.74, 1.28	0.872			
SA	0.97	0.66, 1.42	0.893			
Blood glucose	1.02	0.99, 1.06	0.181			
Creatinine	1.004	1.001, 1.007	0.004	1.00	1.00, 1.01	0.106
Uric acid	1.001	0.999, 1.002	0.075	1.00	1.00, 1.00	0.335
Bilirubin	1.009	0.996, 1.023	0.161			
HDL-C	0.91	0.63,1.31	0.595			
TG	0.99	0.90, 1.07	0.775			
TC	1.01	0.91, 1.13	0.788			
LDL-C	1.06	0.94, 1.20	0.317			
Aspirin	1.52	0.46,5.07	0.493			
Clopidogrel	0.78	0.46,1.34	0.375			
Statin	0.82	0.53,4.31	0.380			
Beta-blocker	0.99	0.77, 1.28	0.956			
ACEI	1.29	1.01, 1.63	0.034	1.20	0.93, 1.54	0.161
CCB	0.95	0.73, 1.24	0.719			
Radial artery access	0.92	0.45,1.92	0.837			
1-vessel disease	0.67	0.52, 0.86	0.001	0.87	0.49, 1.58	0.640
2-vessel disease	0.85	0.67, 1.09	0.201			
3-vessel disease	1.92	1.50, 2.46	<0.001	1.25	0.69, 2.20	0.446
CTO	2.35	1.68,3.28	<0.001	1.84	1.25, 2.68	0.002
Occulsion	1.23	0.89,1.70	0.196			
Bifurcation lesion	0.91	0.67,1.23	0.550			
Restenosis	1.77	0.74, 4.17	0.194			
Prior CABG	0.00001	0.00000, Inf	0.975			
Location of target lesions						
LM	1.47	0.82, 2.62	0.195			
LAD	1.49	1.06, 2.07	0.020	1.22	0.69, 2.24	0.497
LCX	1.34	1.07, 1.69	0.012	0.88	0.51, 1.55	0.642
RCA	1.48	1.17, 1.86	<0.001	1.02	0.60, 1.79	0.933
Number of treated vessels						
1	-	-				
2	0.96	0.74, 1.24	0.774	0.67	0.46, 0.97	0.035
≥3	2.13	1.50, 3.03	<0.001	1.22	0.70, 2.10	0.478
Diameter of stents	0.60	0.45, 0.79	<0.001	0.74	0.54, 0.999	0.048
Length of stents	1.007	1.003, 1.010	<0.001	1.00	1.00, 1.01	0.274
Type of stents						
Sirolimus-eluting stent						
Paclitaxel-eluting stent	0.88	0.64, 1.19	0.419	1.02	0.73, 1.40	0.920
Other drug-eluting stents	1.31	0.96, 1.78	0.086	1.09	0.76, 1.53	0.644

Results were shown as OR(95%CI).

Multivariable analysis module adjust for age, HF, AF, OMI, stroke, prior PCI, DM, SBP, creatinine, uric acid, ACEI, 1-vessel disease, 3-vessel disease, CTO, LAD, LCX, RCA, number of treated vessels, type of stents, diameter of stents, length of stents.

Abbreviations as in **Table 1**.

### 3.3 Risk factors for a single event of MACCEs

Univariable logistic regression analysis showed that the AIP (per 1 SD increase) was a risk factor for TVR (OR = 1.25, 95% CI = 1.03–1.51, *p* = 0.025). After adjusting for confounders (age, HF, AF, OMI, stroke, prior PCI, DM, SBP, creatinine, UA, ACEI, 1-vessel disease, 3-vessel disease, CTO, LAD, LCX, RCA, number of treated vessels, type of stents, diameter of stents, length of stents), the AIP (per 1 SD increase) remained an independent risk factor for TVR in the multivariable logistic regression model (OR = 1.27, 95% CI = 1.02–1.56, *p*= 0.029). However, no association was found between the AIP and all-cause death, AMI, and stroke (**Table 3**).

**Table 3:** Association between AIP (per 1 SD increase) and single event of endpoint.

	Univariate analysis			Multivariate analysis		
	OR	95% CI	p-value	OR	95% CI	p-value
All-cause death	0.87	0.74, 1.02	0.077	0.93	0.77, 1.12	0.425
AMI	0.99	0.81, 1.21	0.933	0.94	0.76,1.17	0.601
Stroke	0.92	0.66, 1.28	0.620	1.08	0.73,1.57	0.707
TVR	1.25	1.03, 1.51	0.025	1.27	1.02,1.56	0.029

Results were shown as OR (95%CI).

Multivariable analysis module adjust for age, HF, AF, OMI, stroke, prior PCI, DM, SBP, creatinine, uric acid, ACEI, 1-vessel disease, 3-vessel disease, CTO, LAD, LCX, RCA, number of treated vessels, type of stents, diameter of stents, length of stents.

Abbreviations as in **Table 1**.

### 3.4 AIP and TVR in the overall population

We categorized all participants into two groups for comparative analysis: the TVR group (n = 106) and the non-TVR group (n = 2,144). The findings revealed that AIP was significantly higher in the TVR group compared to the non-TVR group (0.27 ± 0.33 vs. 0.20 ± 0.29, *p* =0.024). Additionally, SBP, DBP, the prevalence of stroke, prior PCI, restenosis, the number of treated vessels, stent diameter, stent length, and stent type were significantly higher in the TVR group (all *p* < 0.05). There were no significant differences between the groups in terms of gender, age, BMI, blood glucose, TG, TC, LDL-C, HDL-C, creatinine, bilirubin, UA, and other medical history (**Supplementary Table S1**).

After excluding TC, TG, and HDL-C due to collinearity, when AIP (per SD increase) was introduced as a continuous variable into the multivariate regression model, the result in model 3 was significant (OR 1.26, 95%CI 1.01–1.58, *p*=0.042). Additionally, when AIP was categorized into tertiles, higher TVR risk was noted in the third tertile of AIP compared to the second tertile (Model 1: OR = 1.65, 95% CI = 1.02-2.70, *p* = 0.043; Model 2: OR = 1.68, 95% CI =1.04-2.75, *p* = 0.037; Model 3: OR = 1.77, 95% CI = 1.04-3.07, *p* = 0.037) (**Table 4**).

**Table 4:** Analysis of the association between AIP (per 1 SD increase) and the incidence of TVR.

	Model 1			Model 2			Model 3		
Characteristic	OR	95% CI	p-value	OR	95% CI	p-value	OR	95% CI	p-value
**AIP (per 1 SD increase)**	1.25	1.03, 1.51	0.025	1.26	1.03, 1.53	0.023	1.26	1.01, 1.58	0.042
Categories									
**Tertile 2**	Ref	Ref		Ref	Ref		Ref	Ref	
**Tertile 1**	1.19	0.71, 2.00	0.503	1.20	0.72, 2.03	0.482	1.31	0.74, 2.32	0.353
**Tertile 3**	1.65	1.02, 2.70	0.043	1.68	1.04, 2.75	0.037	1.77	1.04, 3.07	0.037

Results were shown as OR(95%CI).

Model 1: Unadjusted.

Model 2: Adjust: age, sex.

Model 3: Adjust: age, sex, SBP, DBP, stroke, prior PCI, CAD,3-vessel disease, restenosis, CTO, bifurcation lesion, number of treated vessels, type of stents, diameter of stents, length of stents, LM, LAD,RCA, LDL-C, DM, smoking, uric acid, blood glucose, AF, OMI, hypertension, aspirin, clopidogrel, and statin.

Abbreviations as in **Table 1**.

### 3.5 Subgroup analysis for the association between the AIP and TVR

**Figure 3:**
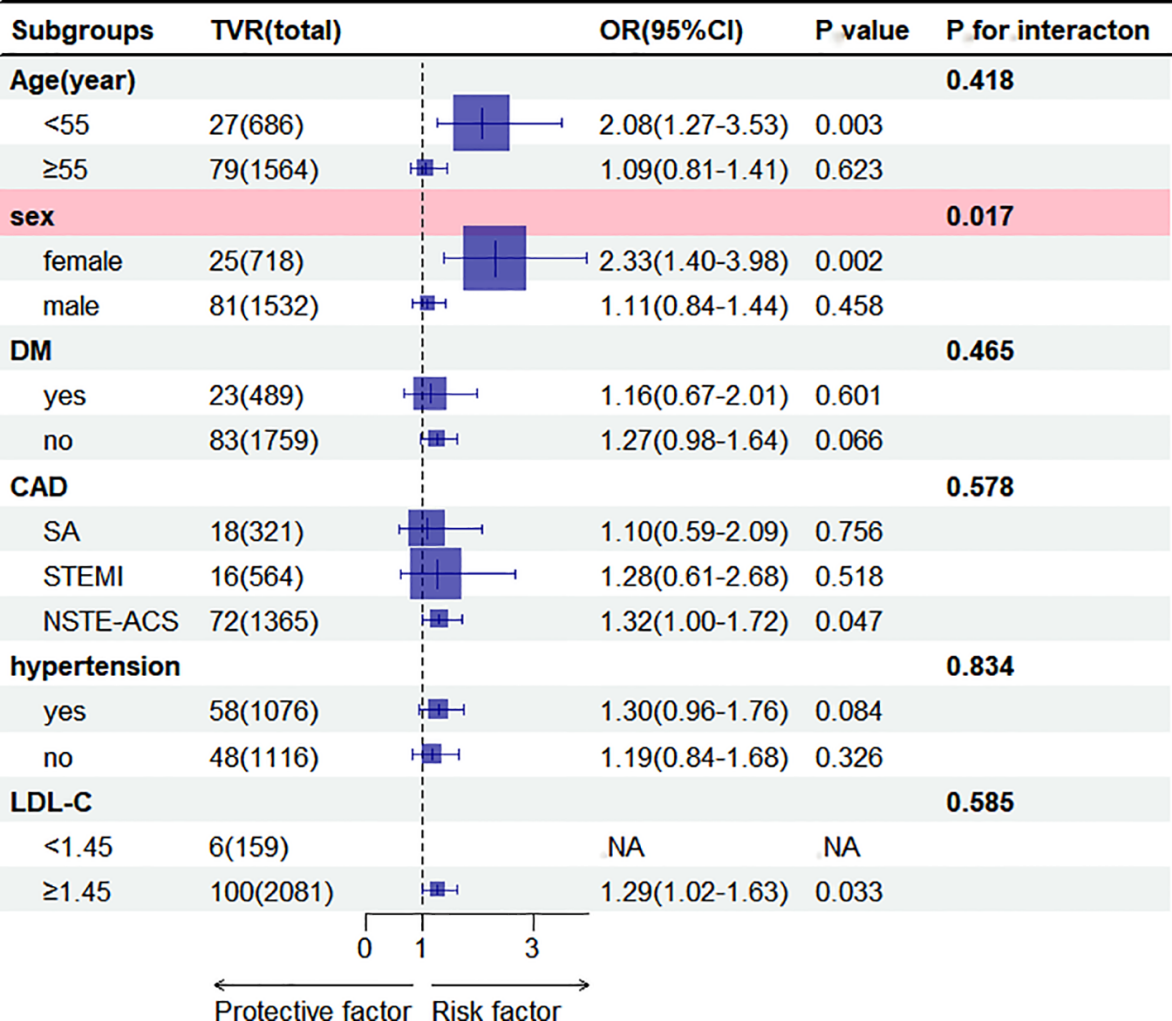
Subgroup analysis of the association between AIP (per 1 SD increase) and TVR. Results were shown as OR (95%CI). Adjustments were made for age, sex, DM, stroke, AF, OMI, hypertension, prior PCI, CAD, smoking, SBP, DBP,3-vessel disease, restenosis, CTO, bifurcation lesion, number of treated vessels, type of stents, diameter of stents, length of stents, LM, LAD,RCA, LDL-C, uric acid, blood glucose, aspirin, clopidogrel, statin in the multivariable model, excluding the strata variables. NA, Not Available. AIP, atherogenic index of plasma; ACS, acute coronary syndrome; CAD, coronary artery disease; DM, diabetes mellitus; LDL-C, low-density lipoprotein cholesterol; SBP, systolic blood pressure; DBP, diastolic blood pressure; LAD, left anterior descending; LM, left main coronary artery; RCA, right coronary artery; AF, atrial fibrillation; OMI, old myocardial infarction; PCI, Percutaneous coronary intervention; CTO, chronic total occlusions; SA, stable angina; TVR, target vessel revascularization. OR, Odds ratio; CI, confidence interval.

Subgroup analysis revealed a significant association between AIP and TVR in females (OR = 2.33, 95% CI = 1.40-3.98, *p* = 0.002), with no significant association in males. An interaction with gender was observed (*p* = 0.017). No other subgroups showed significant interactions (**Figure 3**).

### 3.6 RCS analysis for the relationship between the AIP and TVR

**Figure 4:**
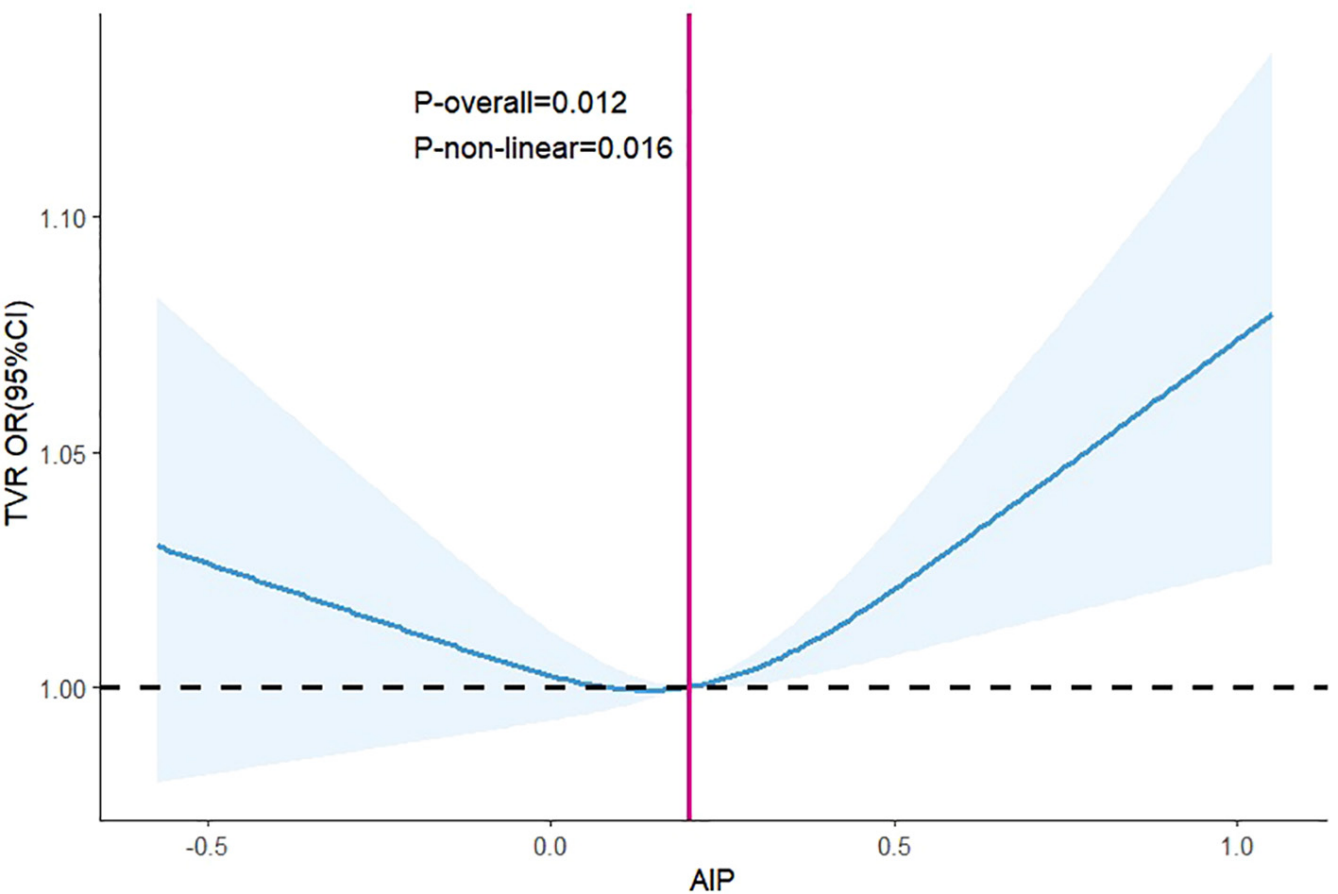
Dose-response between AIP and the odds ratio of TVR. Results were shown as OR (95%CI). Age, sex, SBP, DBP, stroke, prior PCI, smoking history, AF, OMI, hypertension, DM, CAD, 3-vessel disease, restenosis, CTO, bifurcation lesions, number of treated vessels, type of stents, the diameter of stents, length of stents, LM, RCA, LAD, LDL-C, uric acid, blood glucose, aspirin, clopidogrel, statin were adjusted. Abbreviations as in **Figure 3**.

RCS analysis demonstrated a nonlinear “U-shaped” relationship between AIP and TVR (*p* = 0.016), with increased risk evident from AIP value of 0.20 (**Figure 4**).

## 4 Discussion

The primary findings of this study were as follows: (1) For patients with CAD who underwent DES implantation, AIP was an independent risk factor for TVR but not significantly linked to MACCEs; (2) The relationship between AIP and TVR was significant in females but not in males, indicating an interaction between AIP and gender; (3) The association between AIP and TVR showed a “U-shaped” pattern, with a tendency toward a consistently higher TVR risk starting from the AIP value of 0.20.

Consistent with the findings of prior studies, the current investigation found that the AIP was independently associated with TVR development after DES implantation in CAD patients. Qin et al. (20) observed a significant link between AIP and MACCEs in 2,556 CAD patients with comorbid DM who underwent PCI and were followed up for 47.5 months; their subgroup analyses revealed that this association originated from the fact that unplanned TVR was significantly higher in the high AIP group. Wang et al. (22) reported that the elevated AIP value was a strong indicator of postoperative TVR in ACS patients who underwent PCI and had a plasma LDL-C level of <1.8 mmol/L. Ma et al. (19) also found an increased risk of unplanned TVR after PCI in patients with ACS combined with DM at a higher AIP value. However, the AIP index of plasma was not significantly linked to all-cause mortality, cardiovascular mortality, nonfatal ischemic stroke, and nonfatal spontaneous myocardial infarction. In contrast, some studies have revealed no significant correlation between the AIP and TVR incidence after PCI. Drwila et al. (23) found that the AIP was not an independent risk factor for MACEs in elderly patients with non-STEMI in 1-year follow-up. Hartopo et al. (26), however, reported that the probability of in-hospital MACEs was higher in AMI patients in the low-level AIP group and that AIP < 0.24 was an independent predictor of in-hospital all-cause mortality in AMI patients. The outcome was probably associated with the fact that the study only included the older population with AMI and the follow-up period was short. The present study did not identify a significant link between the AIP and MACCEs after PCI in patients with CAD, even after adjusting for confounding variables in the multivariable regression analysis. Additionally, the all-cause mortality rate was higher in the low-level AIP group, although the difference was not statistically significant. This might be due to the low occurrence of MACCEs at 15% and a short follow-up period in the present study.

The current study used RCS to examine the nonlinear correlation between the AIP and TVR after pharmaceutical stenting in CAD patients. The finding indicated a “U-shaped” correlation between the AIP and TVR, with a key point around 0.20 when the risk of TVR notably rose. The conclusion was similar to those reported by Zheng (21), who found that the relationship between the AIP and the risk of MACCEs after PCI in CAD patients without comorbid DM showed a change in the “J-shaped” curve and that the risk of MACCEs was significantly elevated when the AIP value was >0.18 after PCI. This “U-shaped” change may be associated with a “U-shaped” change in the HDL-C level and MACEs (27). HDL-C is often identified as a preventive element in several previous cardiovascular event risk prediction models and is referred to as “good cholesterol.” Nevertheless, a recent study revealed that the relationship between elevated HDL-C levels and the risk of death in CAD patients changed into a “U-shaped” curve, with higher HDL-C levels associated with a higher risk of death (28). In the general population, a “U-shaped” relationship was observed between HDL-C level and stroke risk, with either low or high HDL-C level associated with an increased risk of stroke (29).

In the comparison of the AIP and the patient population in prior studies, gender differences were noted in the risk of cardiovascular diseases and poor prognosis (12, 30). The current investigation found that the AIP was linked to TVR in the female subgroup but not in the male cohort. We found an interaction between the AIP and gender, with the occurrence of elevated AIP levels in female patients at a higher risk of developing TVR. Two primary causes might explain this phenomenon: (1) female patients had a higher mean age compared to male patients (63.1 vs. 58.6 years), and (2) the female subgroup had a larger proportion of postmenopausal women. Elderly women often exhibit a loss of the protective effects of estrogen on the cardiovascular system and insulin sensitivity, leading to a higher likelihood of dyslipidemia, endothelial dysfunction, and insulin resistance (31). Consequently, this elevates the chance of a negative outcome for elderly women. Bendzala et al. (32) also reported a high AIP level as a predictor of all-cause mortality in elderly women. Moreover, AIP and the risk of TVR was positively and independently correlated in patients with LDL-C≥1.45 mmol/L in subgroup analysis, which suggested that lipid-lowering therapy post-PCI, particularly targeting low-density lipoprotein cholesterol (LDL-C), was crucial for improving long-term patient outcomes. A study retrospectively evaluated 1193 *de novo* lesions in 720 patients revealed that lowering the LDL-C level using statins was more effective for preventing late target lesion revascularization after everolimus-eluting stent implantation (33). A meta-analysis included six randomized clinical trials comprising 2,979 patients reported that post-PCI statin therapy was associated with a significantly decreased risk of TVR (34). The observed beneficial effects on TVR may be explained by the pleiotropic effects of statins. Lipid-lowering therapy with statins not only decreased LDL-C and TG, but also increased HDL-C, thus significantly reducing AIP. And Statin therapy also has been shown to inhibit platelet aggregation and the release of platelet derived mediators, as well as to reduce inflammatory responses of the vascular wall, and to improve coronary endothelial function, which all may contribute to the beneficial effect of statin therapy on proliferative responses after coronary stent implantation in humans (35, 36). Additionally, not exactly consistent with previous studies (19, 22, 37), the relationship between AIP and TVR was mainly observed in the non-STEMI population. These inconsistent results on the association may be attributable to the sample size, occurrence rate of TVR, medical level, the length of follow-up, the method of statistical analysis, the adjusted covariables, environment, or other factors.

In-stent restenosis and in-stent thrombosis were the direct causes of unplanned TVR after PCI (38). Zhu et al. (37) identified AIP as an independent risk factor for in-stent restenosis after PCI. Özcan et al. (39) reported that AIP could function as an independent predictor of in-stent thrombosis. The endothelialization process of excessive intimal hyperplasia after DES implantation is often accompanied by *de novo* plaque formation (40). Furthermore, the increased AIP facilitated coronary plaque formation, hastening the progression of atherosclerosis (17). The possible mechanism was as follows. First, the AIP responded to the diameter of small and dense low-density lipoprotein (sdLDL) particles, i.e., the higher the AIP value, the smaller the diameter of the sdLDL particles. The small size and large number of sdLDL particles increased their surface area. Moreover, the diminished salivary acid content of sdLDL particles made it easier for them to cross the vascular endothelium and bind to glycoproteins in the arterial wall, thereby gradually resulting in the deposition of lipids and the initiation of the atherosclerotic process (41). Second, sdLDL was easily oxidized to form oxidized LDL-C and oxidized LDL-C triggered the aggregation of chemokines and adhesion molecules, thereby inducing the transformation of monocytes into macrophages to produce a large number of foam cells under the action of cholesterol, finally leading to atherosclerosis (42). Third, the AIP was closely linked to insulin resistance, which led to decreased vascular endothelial function and increased systemic vascular inflammation, speeding up coronary atherosclerosis (43). Insulin resistance was a significant predictor of MACCEs after coronary pharmacological stenting (44).

## 5 Strengths and limitations

This investigation boasted several notable strengths. To the best of our knowledge, this study was the first to explore the relationship between AIP and TVR in Chinese patients who underwent PCI with DES. Moreover, this study exclusively included patients treated with DES, all of them completed the follow-up. The insights derived from this research could provide valuable guidance for reducing TVR following DES implantation.

However, the current study was subject to several limitations that warrant consideration. First, as a single-center retrospective cohort study, the findings primarily indicated a correlation between the AIP and TVR, without establishing causality. The robustness of this association may be weakened by absent data and uncontrolled confounding factors inherent in the original study design. Second, a notable proportion of the cohort, approximately 11.4%, opted for pharmacological conservative management for recurrent angina during the follow-up period, and only 23.8% underwent repeat coronary angiography. This underrepresentation in diagnostic follow-up may lead to an underestimation of MACCEs, particularly TVR, relative to prior reports. Third, critical parameters such as TG and HDL-C were only measured at baseline and not monitored throughout the follow-up period to assess changes in AIP. Variations in these parameters could be influenced by changes in dietary habits, physical activity, and medication adherence, factors which were not accounted for in this study.

## 6 Conclusions

In summary, the AIP was a significant predictor of TVR after the implantation of DES in patients with CAD, particularly in female patients. A “U-shaped” relationship curve was observed between the AIP value and TVR after DES implantation. Based on these findings, we suggested that the AIP should be used as a plasma marker of key interest for preventing TVR after DES implantation in patients with CAD. Multicenter prospective studies with larger sample sizes are required to further confirm these findings.

## Data Availability

The original contributions presented in the study are included in the article/**Supplementary Material**. Further inquiries can be directed to the corresponding author.
