# Supplementary material for: A U-shaped relationship between the atherogenic index of plasma and repeated target vessel revascularization in patients undergoing percutaneous coronary intervention: a retrospective study
Source: Front Endocrinol (Lausanne). 2024 Nov 11;15:1428830. doi: 10.3389/fendo.2024.1428830 (PMC11586652; doi:10.3389/fendo.2024.1428830)
Supplement: Supplementary file 1 [file Table1.docx]

Table S1 Baseline characteristics of the study population according to TVR tertiles

| Characteristic | Overall, N = 2,250 | Non-TVR, N = 2,144 | TVR, N = 106 | *p*-value |
| --- | --- | --- | --- | --- |
| Demographics |  |  |  |  |
| Age, years | 60.0±11.1 | 60.0±11.1 | 60.2±10.1 | 0.827 |
| Male, n (%) | 1,532 (68%) | 1,451 (68%) | 81 (76%) | 0.060 |
| BMI, kg/m^2^ | 23.9±3.8 | 23.9±3.8 | 23.5±3.5 | 0.497 |
| SBP, mmHg | 103±29 | 102±29 | 115±31 | <0.001 |
| DBP, mmHg | 77±12 | 77±12 | 80±13 | 0.046 |
| Medical history |  |  |  |  |
| HF, n(%) | 269 (12%) | 254 (12%) | 15 (14%) | 0.480 |
| AF, n(%) | 44 (2.0%) | 40 (1.9%) | 4 (3.8%) | 0.150 |
| OMI, n(%) | 221 (9.8%) | 209 (9.7%) | 12 (11%) | 0.595 |
| Stroke, n(%) | 118 (5.2%) | 107 (5.0%) | 11 (10%) | 0.015 |
| Prior PCI, n(%) | 149 (6.6%) | 134 (6.3%) | 15 (14%) | 0.001 |
| Prior CABG, n(%) | 16 (0.7%) | 16 (0.7%) | 0 (0%) | >0.999 |
| Hypertension, n(%) | 1,133 (50%) | 1,075 (50%) | 58 (55%) | 0.360 |
| DM, n(%) | 489 (22%) | 466 (22%) | 23 (22%) | 0.990 |
| Smoking, n(%) | 740 (33%) | 702 (33%) | 38 (36%) | 0.506 |
| CAD |  |  |  | 0.051 |
| STEMI, n (%) | 564 (25%) | 548 (26%) | 16 (15%) |  |
| NSTE-ACS, n (%) | 1,365 (61%) | 1,293 (60%) | 72 (68%) |  |
| SA, n(%) | 321 (14%) | 303 (14%) | 18 (17%) |  |
| Laboratory data |  |  |  |  |
| Blood glucose (mmol/L) | 5.2 (4.7, 6.3) | 5.2 (4.7, 6.3) | 5.3 (4.6, 6.4) | 0.866 |
| Creatinine (µmol/L) | 69.0 (58.0, 81.0) | 69.0 (58.0, 81.0) | 71.5 (61.0, 83.0) | 0.093 |
| UA (µmol/L) | 294.0 (244.0, 353.0) | 295.0 (244.0, 354.0) | 290.0 (243.8, 347.5) | 0.860 |
| Bilirubin (µmol/L) | 8.6 (6.0, 12.0) | 8.6 (6.0, 12.0) | 8.9 (6.8, 12.6) | 0.170 |
| HDL-C (mmol/L) | 1.1±0.3 | 1.1±0.3 | 1.0±0.3 | 0.509 |
| TG (mmol/L) | 1.6 (1.1, 2.2) | 1.6 (1.1, 2.2) | 1.7 (1.2, 2.7) | 0.061 |
| TC (mmol/L) | 4.3±1.1 | 4.3±1.1 | 4.4±1.1 | 0.227 |
| LDL-C (mmol/L) | 2.7±0.9 | 2.67±0.9 | 2.71±0.9 | 0.626 |
| AIP | 0.20±0.29 | 0.20±0.29 | 0.27±0.33 | 0.024 |
| AIP ≥ 0.314(Tertile 3), n(%) | 751 (33%) | 706 (33%) | 45 (42%) | 0.042 |
| Treatment |  |  |  |  |
| Aspirin, n(%) | 2,220 (99%) | 2,115 (99%) | 105 (99%) | 1 |
| Clopidogrel, n(%) | 2,158 (96%) | 2,057 (96%) | 101 (95%) | 0.228 |
| Statin, n(%) | 2,111 (94%) | 2,015 (94%) | 96 (91%) | 0.154 |
| Beta blocker, n(%) | 1,583 (70%) | 1,508 (70%) | 75 (71%) | 0.926 |
| ACEI, n(%) | 1,248 (55%) | 1,195 (56%) | 53 (50%) | 0.244 |
| CCB, n(%) | 557 (25%) | 529 (25%) | 28 (26%) | 0.685 |
| Radial, artery access, n(%) | 2,195 (98%) | 2,090 (97%) | 105 (99%) | 0.517 |
| Number of diseased vessels, n(%) |  |  |  |  |
| 1-vessel disease | 877 (39%) | 843 (39%) | 34 (32%) | 0.136 |
| 2-vessel disease | 836 (37%) | 797 (37%) | 39 (37%) | 0.937 |
| 3-vessel disease | 532 (24%) | 499 (23%) | 33 (31%) | 0.063 |
| Characteristics of lesions |  |  |  |  |
| CTO, n(%) | 197 (8.8%) | 185 (8.6%) | 12 (11%) | 0.338 |
| Occulsion, n (%) | 296 (13%) | 286 (13%) | 10 (9.4%) | 0.246 |
| Bifurcation lesion, n(%) | 396 (18%) | 374 (17%) | 22 (21%) | 0.382 |
| Restenosis, n(%) | 29 (1.3%) | 25 (1.2%) | 4 (3.8%) | 0.045 |
| Location of target lesions |  |  |  |  |
| LM, n (%) | 72 (3.2%) | 67 (3.1%) | 5 (4.7%) | 0.386 |
| LAD, n(%) | 1,859 (83%) | 1,770 (83%) | 89 (84%) | 0.709 |
| LCX, n (%) | 1,096 (49%) | 1,038 (48%) | 58 (55%) | 0.205 |
| RCA, n (%) | 1,109 (49%) | 1,048 (49%) | 61 (58%) | 0.081 |
| CABG, n(%) | 2 (<0.1%) | 2 (<0.1%) | 0 (0%) | >0.999 |
| Number of treated vessels |  |  |  | <0.001 |
| 1 | 1,293 (57%) | 1,245 (58%) | 48 (45%) |  |
| 2 | 757 (34%) | 719 (34%) | 38 (36%) |  |
| ≥3 | 200 (8.9%) | 180 (8.4%) | 20 (19%) |  |
| Diameter of stents (mm) | 3.10±0.91 | 3.10±0.93 | 2.95±0.43 | 0.001 |
| Length of stents (mm) | 42.0 (24.0, 66.0) | 41.0 (24.0, 66.0) | 51.0 (33.0, 80.0) | 0.006 |
| Type of stents |  |  |  | 0.008 |
| Sirolimus-eluting stent, n (%) | 1,464 (65%) | 1,404 (66%) | 60 (57%) |  |
| Paclitaxel-eluting stent, n(%) | 443 (20%) | 425 (20%) | 18 (17%) |  |
| Other drug-eluting stents, n (%) | 339 (15%) | 312 (15%) | 27 (26%) |  |
